# Violence Victimization in Korean Adolescents: Risk Factors and Psychological Problems

**DOI:** 10.3390/ijerph14050541

**Published:** 2017-05-19

**Authors:** Subin Park, Yeeun Lee, Hyesue Jang, Minkyung Jo

**Affiliations:** 1 Department of Research Planning, Mental Health Research Institute, National Center for Mental Health 127, Yongmasan-ro, Gwangin-gu, Seoul 04933, Korea; hsjang0315@gmail.com (H.J.); jomk@korea.kr (M.J.); 2 Department of Psychology, Korea University, 145 Anam-ro, Seongbuk-gu, Seoul 02841, Korea; tasarang1010@gmail.com

**Keywords:** risk factors, violence, victim, adolescents, bullying

## Abstract

We examined the risk factors for and psychological problems associated with violence victimization in a nationwide representative sample of Korean adolescents. Data from the 2016 Korean Youth Risk Behavior Web-based Survey was used. Participants were asked about their experience of being a victim of violence that required medical treatment during the past 12 months, as well as their perceived health, happiness, sleep satisfaction, stress, depressed mood, and suicidality. The 12-month prevalence of violence victimization requiring medical treatment was 2.4%. The results indicated that adolescents were at an increased risk for violence victimization if they were male, older, had parents of a foreign nationality, did not reside with their family, worked part time, resided in small cities or rural areas, were high or low in socioeconomic status (SES), exhibited high or low levels of academic performance, used alcohol or tobacco, and were sexually active. In addition, while violence victimization was negatively associated with perceived health and happiness, it was positively associated with perceived stress, depressed mood, and suicidality. The results indicate that a social disadvantage, involvement in risky behavior, and psychological problems are associated with violence victimization. Effective violence prevention efforts should thus target high-risk groups, and clinical attention is needed to address the psychological costs associated with violence victimization.

## 1. Introduction

Research suggests that more than half of the world’s children and adolescents have experienced some form of violence in the past 12 months, with Asian countries having the highest number of children and adolescents exposed to such violence [1]. Violence victimization includes experiences of physical, sexual, and emotional violence [2], and it is a key risk factor for mental health problems and suicide among adolescents, with psychological manifestations extending into adulthood [3,4,5]. Despite its high prevalence, violence victimization should not be regarded as a normative aspect of development [6], considering the long-lasting and adverse impact it has on its victims in terms of social and psychological costs [7].

Violence victimization among adolescents is linked to a wide range of psychiatric symptoms, such as depression, anxiety, and suicidality [8,9,10,11]. In particular, experiencing prior victimization at an early age has been found to predict an increased risk for depression or deviance later in life [5,12,13]. Moreover, according to longitudinal studies, involvement in violence is stable across time, such that victims are highly likely to remain victims, even after several years [14,15,16]. Therefore, it is crucial to identify the risk factors for violence victimization among adolescents in order to provide high-risk groups with timely interventions that mitigate its long-term effects.

Research has shown that there is a wide spectrum of risk factors associated with violence victimization, including social, familial, and individual variables. For example, individuals whose environments are characterized by socioeconomic disadvantage, such as parental maltreatment, a non-intact family structure, and low socioeconomic status, are at an increased risk for victimization [9,17,18,19,20,21]. With regard to individual factors, boys are more likely to be involved in violence, and several aspects of physical appearance, including physical weakness, obesity, and small stature, have also been found to increase the risk for victimization [16,22]. In addition, youth with internalizing problems [23,24] or who lack social competence are at an increased risk of being victimized by their peers. By simultaneously considering environmental and individual variables, Veenstra et al. [25] has argued that environmental factors may influence the risk of victimization through changes in personal characteristics, such as unlikability or aggressiveness.

Among Korean adolescents, violence is a serious health concern. In particular, 30 to 60% of Korean youth have reported being involved in violence such as bullying, either by engaging in the act of bullying or by being bullied themselves. In addition, the prevalence of victimization ranges from 23 to 64% [23,26,27]. However, to date, only a few studies have investigated the risk factors for violence victimization among youth in Korea [26]. To enhance our understanding of these risk factors, the present study examined the associations between violence victimization and a wide range of environmental, familial, and socio-demographic variables in a national representative sample of Korean adolescents. This study further examined the associations between adolescent risk behaviors and psychiatric symptoms to understand the potential mental health outcomes of victimization.

## 2. Methods

### 2.1. Subjects

The Korea Youth Risk Behavior Web-based Survey (KYRBS) is an anonymous online self-report survey that has been conducted annually since 2005 by the Korea Centers for Disease Control and Prevention. The KYRBS seeks to identify teenagers’ health behaviors among those who range from middle school freshmen to high school seniors. The current study used stratified cluster sampling to extract middle and high school student samples that were representative of the teenage population. After survey instructions were provided, students who agreed to participate in the study completed the survey during class. Personal data, including respondents’ names, schools, phone numbers, addresses, and resident registration numbers, were not collected. In 2015, the survey included a total of 800 schools, including 400 middle schools and 400 high schools, and a total of 65,528 respondents participated, resulting in a 96.4% participation rate. The average age of the respondents was 14.99 years (±1.74), with respondents ranging in age from 12 to 18 years. Given that details regarding the current sampling and survey methods can be easily obtained from other sources, it is not discussed in additional detail here [28,29]. The KYRBS was approved by the institutional review board of Korea Centers for Disease Control and Prevention (2014-06EXP-02-P-A).

### 2.2. Measures

Violence victimization that required medical treatment was assessed via the following question: “Have you been a victim of physical violence, threats of violence, or bullying that required medical or clinical treatment during the past 12 months?”

Sociodemographic variables included gender, age, place of residence (e.g., rural area, small city, or large city), residence type (e.g., with immediate family, with relatives, with friends, alone, in a dormitory, or in a facility), parental birthplace (e.g., South Korea or foreign country), presence or absence of a part-time job, perceived socioeconomic status (SES), and perceived academic performance. Perceived SES and academic performance were assessed on a five-point Likert scale (e.g., low (1), low-middle (2), middle (3), high-middle (4), and high (5)) and were classified into three groups for logistic regression analyses: low (1), middle (2–4), and high (5) [28,29].

Perceived body shape was assessed on a five-point Likert scale and was classified into three groups: very underweight (1), normal weight (2–4), and very overweight (5). Lifetime alcohol and tobacco use was assessed with the following questions: “Have you ever used alcohol?” and “Have you ever used tobacco?” Lifetime sexual intercourse was assessed with the following questions: “Have you ever engaged in sexual intercourse with the opposite sex?” and “Have you ever engaged in sexual intercourse with the same sex?” Forced-choice “yes” or “no” response options were given for these questions [28].

Participants’ degree of perceived health was evaluated with the following question: “How healthy do you usually feel?”, with participants choosing one of the following response options: (1) very healthy, (2) healthy, (3) average, (4) unhealthy, or (5) very unhealthy. Based on their answers, respondents were categorized into one of two groups: an above average health group (1–2) and an average or below average health group (3–5). Participants’ degree of perceived happiness was evaluated with the following question: “How happy do you usually feel?”, with participants choosing one of the following response options: (1) very happy, (2) happy, (3) average, (4) unhappy, or (5) very unhappy. Based on their answers, respondents were categorized into one of two groups: an above average happiness group (1–2) and an average or below average happiness group (3–5). The degree of sleep satisfaction was evaluated with the following question: “In the past seven days, did you get adequate sleep to overcome fatigue?”, with participants choosing from one of the following response options: (1) very adequate, (2) adequate, (3) average, (4) not adequate, or (5) not very adequate. Based on their answers, respondents were categorized into one of two groups: an above average sleep satisfaction group (1–2) and an average or below average sleep satisfaction group (3–5). The degree of perceived stress was measured with the following question: “To what degree are you usually stressed?”, with participants choosing from one of the following response options: (1) very much, (2) somewhat, (3) average, (4) not so much, or (5) not at all. Based on their answers, respondents were categorized into one of two groups: an above average stress group (1–2) and an average or below average stress group (3–5) [28,29].

Participants’ signs and symptoms of depression in the past 12 months were measured with the following question: “Have you experienced sadness or despair to the degree that you stopped your daily routine for two weeks?” Suicidal ideation, planning, and attempts were evaluated with the following questions, respectively: “In the past 12 months, have you ever thought of committing suicide?”, “Have you planned suicide in detail?”, and “Have you ever attempted suicide?” Forced-choice “yes” or “no” response options were given for these questions [28,29].

### 2.3. Statistical Analysis

Descriptive statistics were first calculated, and logistic regression analyses were subsequently conducted to compare socio-demographic and behavioral factors between victims of violence and non-victims. Odd ratios (ORs) and 95% confidence intervals (CIs) were calculated using 12-month violence victimization as the main outcome variable, and each socio-demographic and behavioral variable as principal predictors. To elucidate the association between violence victimization and psychological problems, logistic regression analyses were conducted using 12-month violence victimization as the principal predictor and each psychological problem as the main outcome variable. These analyses were conducted both with and without socio-demographic and behavioral covariates that significantly differed between groups. SAS (version 8.5, SAS Institute Inc., Cary, NC, USA) [30] was used to perform all statistical analyses, and a *p*-value less than 0.05 was considered significant. All analyses were conducted to consider the sample design, population parameters, weighting, clustering, and stratification. The rate of post-hoc weight adjustment was calculated as the sum of the weights of gender, type of school, and grades to proportionally represent the student population among national middle and high schools in April 2016 [28,29].

## 3. Results

Among 65,528 respondents, 1508 (weighted prevalence 2.4%) experienced violence victimization that required medical treatment. Several socio-demographic variables were associated with an increased risk for violence victimization, including being male (OR = 2.19) and having a father and mother of foreign origin (ORs = 17.42 for father, and 4.11 for mother). Similarly, residing in a facility (OR = 25.80), residing with relatives (OR = 3.50), or residing with friends, alone, or in a dormitory (OR = 16.86), were associated with an increased risk for violence victimization compared to residing with family. Lastly, being high or low in SES (socioeconomic status), compared to being in the middle class, also served as a risk factor (ORs = 3.36 and 5.55 for high and low SES, respectively). With regard to individual variables, having a part-time job (OR = 2.97), exhibiting high or low levels of academic performance compared to average levels (ORs = 2.38 and 2.26 for high and low levels, respectively), and being underweight or overweight, compared to being a normal weight (ORs = 2.03 and 1.38, respectively), were associated with an increased risk of experiencing violence victimization. With regard to behavioral variables, using alcohol or tobacco (ORs = 2.10 and 3.56, respectively) and engaging in sexual relations with the opposite sex or the same sex (ORs = 7.86 and 27.48, respectively) were associated with an increased risk for violence victimization (Table 1).

In terms of psychological variables, violence victimization was negatively associated with perceived health (OR = 0.78, 95% CI = 0.70–0.87) and happiness (OR = 0.61, 95% CI = 0.55–0.67). In contrast, violence victimization was positively associated with perceived stress (OR = 1.62, 95% CI = 1.46–1.81), a depressed mood (OR = 3.68, 95% CI = 3.31–4.08), suicidal ideation (OR = 4.61, 95% CI = 4.14–5.14), suicide planning (OR = 8.65, 95% CI = 7.54–9.92), and suicide attempts (OR = 10.92, 95% CI = 9.40–12.68) during the past 12 months. These associations remained significant even after controlling for gender, residence type, SES, parental nationality, part-time job status, academic achievement, alcohol and tobacco use, and experience of sexual intercourse (Table 2). Violence victimization was not significantly associated with sleep satisfaction before (OR = 1.04, 95% CI = 0.93–1.17) and after (OR = 1.04, 95% CI = 0.92–1.18) controlling the covariates.

## 4. Discussion

The present study investigated the association between violence victimization and a wide spectrum of risk factors and psychological problems among Korean adolescents. Our findings demonstrate that gender, a family environment, academic achievement, and perceived body shape are significantly associated with violence victimization. Furthermore, adolescents who had been victims of violence during the past year were more likely to experience negative mental health outcomes compared to those who had not, including risky behavior, depressive symptoms, and suicidality. Our findings indicate that violence victimization is an urgent public mental health concern for Korean youth.

In addition, the current study found that being male was associated with an increased risk of victimization, which is consistent with prior findings that boys are more likely to be involved in school bullying [4,26,27]. Boys may participate in overt violent behavior as a means by which to exert their dominance and demonstrate their position in the peer group hierarchy. Compared to girls, engaging in this type of behavior is more heavily emphasized among boys [31].

Previous studies have demonstrated that a family environment is strongly associated with victimization risk [21,25]. Similar to past research, the current study found that an individual’s risk for victimization differs as a function of the living arrangement. Namely, youth residing with a relative or in a facility had an approximately 16 to 25 times greater risk of being a victim compared to those who resided with their immediate family. These findings are consistent with prior research regarding the significance of the family structure [26]. This association between the living arrangement and victimization risk may exist due to the maltreatment that youth living without family more often experience [32]. Further, when children are separated from their primary caretaker, who is most often one of their parents, this can prevent them from developing a secure attachment to others and can impact their overall emotional and social development [33,34].

An additional family variable associated with the risk of violence victimization involved SES (socioeconomic status), as has been suggested by previous studies [17]. More specifically, our findings indicate that being high or low in SES was associated with an increased risk for violence victimization, compared to individuals who were in the middle class. This association between low SES and victimization is consistent with prior findings [18,21]. With regard to our findings regarding low SES, Tippett and Wolke [17] suggest that there are two possible mechanisms that explain its association with victimization risk: a lack of access to intellectual resources, as well as poor family functioning. Intellectual resources including norms, values, and problem solving skills can help children to develop social skills and coping strategies, and children’s relationships at home with parents and siblings shape how they interact with peers [17]. In contrast, there are inconsistencies across the literature with regard to the relationship between high SES and violence among adolescents. While the association between high SES and an increased victimization risk is consistent with a study by Kim et al. [26] on Korean adolescents, these findings contradict a meta-analysis conducted by Tippett and Wolke [17], which found that victims are less likely to come from high SES backgrounds. Given these inconsistencies, it may be possible that having a different SES background than one’s peer groups—as opposed to being in a particular SES group per se—may increase an adolescent’s risk for being the target of violence among Korean adolescents, particularly given that violence can be a reaction to deviance [35].

In addition, parental nationality was significantly associated with the risk for victimization among Korean adolescents. Adolescents whose parents originally came from a foreign country were at a greater risk of experiencing victimization compared to their peers. One possible explanation for these findings is that bi-ethnic adolescents in Korea are often exposed to ethnic discrimination, particularly from their peers [36,37]. Having a different appearance and coming from a different family background may cause them to appear conspicuous to their peer groups, particularly in a non-diverse environment such as Korea, therefore increasing their risk of becoming a target of violence.

The current study also found that adolescents who were either high or low in academic achievement were at an increased risk for victimization. Although the association between victimization and low academic achievement has already been demonstrated in a meta-analysis by Nakamoto and Schwartz [38], its association with high academic achievement warrants further investigation. As previously mentioned, students who behave differently than their average peers may be easy targets of violence. In that same vein, adolescents whose weight differed from that of the average peer—either in terms of being underweight or overweight—were also found to have an increased risk for victimization. These findings are consistent with prior research demonstrating that being overweight [22] or being physically weak [24] may increase adolescents’ risk for being victimized.

Furthermore, our findings show that adolescents involved in part-time work are at a greater risk for victimization. To date, studies have found an association between part-time work and problem behaviors among Korean adolescents, such as substance abuse, delinquency, and poor school adaptation [39,40]. Similarly, part-time work may adversely influence an adolescent’s peer relationships. It is possible that the part-time work environment will expose adolescents to risky behaviors, such as drinking and smoking, and estrange them from the school environment and their peer groups [41]. Alternatively, this association might be attributable to the fact that adolescents who engage in part-time work may be more likely to come from low SES and otherwise disadvantaged backgrounds. However, our findings cannot provide a clear causal explanation for this association.

In addition, the current study found that both alcohol and tobacco use increase the risk for victimization among adolescents. These results are consistent with previous findings that have found a direct association between violent victimization at school, elevated alcohol use during the past 12 months, and episodic heavy drinking [42]. Similarly, previous studies have also demonstrated that smokers are more likely to experience violence victimization [43,44]. However, other studies have found that tobacco use is more strongly associated with violence assertion, as opposed to victimization [45,46]. Nevertheless, our results indicate that policies and programs aimed at reducing violence victimization should be directed toward the prevention of alcohol and tobacco use among adolescents.

In terms of risky behaviors, our results also show that adolescents who engaged in sexual relations had a higher chance of becoming a victim of violence, and these results are consistent with previous findings [47,48]. Importantly, adolescent sexual orientation differentially affected adolescents’ risk of being a victim of violence. Namely, students who engaged in same-sex intercourse exhibited an approximate 28-fold increase in their risk for violence victimization compared to students who had never engaged in sexual intercourse. Indeed, previous studies have found that non-heterosexual youth are more susceptible to victimization compared to heterosexual youth [49], with this risk being even higher for girls than boys [47,50]. Though the risks were not as high as they were for non-heterosexual youth, sexually active heterosexual youth were nonetheless still at an increased risk of about eight-fold for victimization compared to students who were not sexually active, which is consistent with previous findings [48].

Furthermore, we found that victims of violence experience several psychological problems, including diminished perceived health and happiness, and increases in perceived stress and a depressed mood. Previous studies have also reported that students who are bullied report diminished physical functioning compared to students who do not experience violence [51]. Politis et al. have investigated the specific symptoms associated with victims of violence among adolescents in Greece and found that victims were more likely to report backache, dizziness, and fatigue, independent of other psychiatric conditions [52]. Moreover, findings from several studies also support the notion that victimization is associated with increased stress [53] and depressive symptoms [54,55,56]. Given that symptoms of depression are more severe among victims who do not seek help [54], violence prevention education should address the importance of seeking help to reduce consequential depression among the victims.

Our results further showed that victims of violence experience an increased suicide risk. Indeed, research conducted with U.S. adolescents has shown that victims of physical violence experience frequent suicidal ideation and attempts [57]. Moreover, a prospective cohort study in South Korea reported that suicidal ideation risk over the course of six months was significantly higher among students who were both victims and perpetrators of violence compared to students who were not involved with violence [58]. Therefore, both public and school-level efforts should be made to protect students involved in violence from suicidality and other adverse consequences.

Overall, our findings suggest that victims of violence are likely to perceive themselves as less healthy and less happy than non-victims. Moreover, victims are at an increased risk for higher stress, a depressed mood, and suicidality (ideation, planning, and attempts) compared to non-victims. Given such findings, we believe that programs related to suicide prevention, stress management, and mental health screening can be helpful for victims of violence to alleviate and manage the psychological problems that they are likely to suffer from.

Although we found evidence of several risk factors for and psychological problems associated with violence victimization, there are some limitations to this study. First, this study failed to include out-of-school youth because the survey was conducted in middle schools and high schools during class times. Out-of-school youth may be at a higher risk for violence victimization because school dropouts are more likely to be exposed to risk factors, such as alcohol and tobacco use [59,60], and be involved in violence [61]. It is also possible that out-of-school youth experience different risk factors for violence victimization than in-school youth [61]. Second, since the study involved the administration of a large self-report survey, we used single item questions to measure the experience of violence victimization, which fails to capture diverse aspects of violence victimization, including its repetitiveness or severity. Lastly, due to the cross-sectional nature of the current study, causality in the association between violence victimization and psychological problems cannot be assumed from the current findings.

Given these limitations, future studies could strengthen the current findings in a few manners. First, future work could include adolescents who are not currently attending school. Moreover, we suggest that future studies use standardized scales to assess violence among adolescents, thereby increasing the construct validity and allowing for an examination of the diverse aspects of violence victimization. Lastly, future studies could adopt prospective designs to investigate the long-term effects of violence victimization and determine the direction of causality between violence victimization and psychological problems, which were not clearly addressed in the current study. The strengths of this study include the use of a large national sample of adolescents in South Korea, as well as an extensive examination of the risk factors for and psychological problems associated with violence victimization.

## 5. Conclusions

Overall, our results indicate that violence victimization among Korean adolescents is associated with social disadvantage, peer-group dissimilarities (e.g., in terms of appearance, nationality), and risky behavior involvement. Violence victimization was further associated with several psychological problems, including a decrease in perceived health and happiness, as well as an increase in stress, a depressed mood, and suicidality. In light of these findings, effective violence prevention efforts should target high-risk groups who exhibit multiple risk factors, and clinical attention is needed to mitigate the psychological consequences of violence victimization.

## Figures and Tables

**Table 1:** Characteristics of violence victims and non-victims among Korean adolescents.

Independent Variables	Non-Victim ^†^ Weighted %	Victim ^‡^ Weighted %	OR (95% CI)
Age, mean (SE)	15.11 (0.02)	15.19 (0.06)	1.03 (0.99–1.06)
Gender, male	51.72	70.11	2.19 (1.93–2.48)
**Area of Residence**			
Large city	43.34	42.80	reference
Small city	50.82	51.08	1.02 (0.88–1.17)
Rural	5.84	6.11	1.06 (0.79–1.42)
**Residence Type**			
With family	95.96	76.74	reference
With relatives	0.60	8.10	16.86 (13.80–20.59)
With friend/alone/in a dormitory	3.13	8.76	3.50 (2.73–4.49)
In a facility	0.31	6.40	25.80 (19.69–33.81)
**Socioeconomic Status**			
High	9.27	23.19	3.36 (2.93–3.84)
Middle	88.01	65.57	reference
Low	2.72	11.25	5.55 (4.71–6.56)
Foreign-born father	0.23	3.34	17.42 (12.93–23.48)
Foreign-born mother	0.94	3.10	4.11 (3.07–5.50)
Having a part-time job	12.39	29.58	2.97 (2.63–3.36)
**Academic Achievement**			
High	12.74	23.27	2.38 (2.08–2.72)
Middle	77.24	59.34	reference
Low	10.02	17.39	2.26 (1.98–2.58)
**Perceived Body Shape**			
Very underweight	4.34	8.25	2.03 (1.69–2.43)
Normal range	89.55	83.86	reference
Very overweigh	6.11	7.89	1.38 (1.13–1.69)
Alcohol use	38.34	56.57	2.10 (1.89–2.32)
Tobacco use	14.30	37.26	3.56 (3.17–3.99)
Sexual relations with the opposite sex	3.64	22.89	7.86 (6.88–8.98)
Sexual relations with the same sex	0.63	14.80	27.48 (23.09–32.72)

^†^ Sample size = 64,020, weighted = 3,110,576; ^‡^ sample size = 1508, weighted = 75,136.

**Table 2:** Associations between violence victimization and psychological problems among Korean adolescents.

Dependent Variables	Non-Victim ^†^ Weighted %	Victim ^‡^ Weighted %	UOR (95% CI)	AOR (95% CI)
Perceived health	71.90	66.58	0.78 (0.70–0.87)	0.76 (0.67–0.86)
Perceived happiness	66.89	55.07	0.61 (0.55–0.67)	0.66 (0.59–0.75)
Sleep satisfaction	25.83	26.60	1.04 (0.93–1.17)	1.04 (0.92–1.18)
Perceived stress	37.08	48.86	1.62 (1.46–1.81)	1.53 (1.35–1.73)
Depressed mood	24.76	54.76	3.68 (3.31–4.08)	3.09 (2.74–3.48)
Suicide ideation	11.50	37.47	4.61 (4.14–5.14)	3.54 (3.11–4.04)
Suicide planning	3.56	24.18	8.65 (7.54–9.92)	4.99 (4.20–5.92)
Suicide attempts	1.99	18.16	10.92 (9.40–12.68)	5.47 (4.50–6.64)

^†^ Sample size = 64,020, weighted = 3,110,576; ^‡^ sample size = 1508, weighted = 75,136; UOR: Unadjusted odds ratio, AOR: odd ratio adjusted for gender, residence type, socioeconomic status, parental birthplace, part-time job status, academic achievement, alcohol and tobacco use, and experience of sexual intercourse.
